# The New Genetic Landscape of Cushing’s Disease: Deubiquitinases in the Spotlight

**DOI:** 10.3390/cancers11111761

**Published:** 2019-11-08

**Authors:** Silviu Sbiera, Meik Kunz, Isabel Weigand, Timo Deutschbein, Thomas Dandekar, Martin Fassnacht

**Affiliations:** 1 Department of Internal Medicine I, Division of Endocrinology and Diabetes, University Hospital, University of Würzburg, D-97080 Würzburg, Germany; weigand_i@ukw.de (I.W.); deutschbein_t@ukw.de (T.D.); fassnacht_m@ukw.de (M.F.); 2 Comprehensive Cancer Center Mainfranken, University of Würzburg, D-97080 Würzburg, Germany; 3 Chair of Medical Informatics, Friedrich-Alexander University of Erlangen-Nürnberg, D-91058 Erlangen, Germany; meik.kunz@fau.de; 4 Department of Bioinformatics, Biocenter, University of Würzburg, D-97074 Würzburg, Germany; dandekar@biozentrum.uni-wuerzburg.de

**Keywords:** Cushing’s disease, pathogenesis, somatic mutations, deubiquitinases

## Abstract

Cushing’s disease (CD) is a rare condition caused by adrenocorticotropic hormone (ACTH)-producing adenomas of the pituitary, which lead to hypercortisolism that is associated with high morbidity and mortality. Treatment options in case of persistent or recurrent disease are limited, but new insights into the pathogenesis of CD are raising hope for new therapeutic avenues. Here, we have performed a meta-analysis of the available sequencing data in CD to create a comprehensive picture of CD’s genetics. Our analyses clearly indicate that somatic mutations in the deubiquitinases are the key drivers in CD, namely USP8 (36.5%) and USP48 (13.3%). While in USP48 only Met415 is affected by mutations, in USP8 there are 26 different mutations described. However, these different mutations are clustering in the same hotspot region (affecting in 94.5% of cases Ser718 and Pro720). In contrast, pathogenic variants classically associated with tumorigenesis in genes like TP53 and BRAF are also present in CD but with low incidence (12.5% and 7%). Importantly, several of these mutations might have therapeutic potential as there are drugs already investigated in preclinical and clinical setting for other diseases. Furthermore, network and pathway analyses of all somatic mutations in CD suggest a rather unified picture hinting towards converging oncogenic pathways.

## 1. Introduction

Cushing’s disease (CD) is caused by pituitary adrenocorticotropic hormone (ACTH) producing adenomas. ACTH oversecretion leads in turn to cortisol overproduction in the adrenal glands that results to the specific clinicopathological manifestations associated with Cushing’s syndrome. The latter is characterized by increased morbidity and mortality, mainly due to cardiovascular impairment [1]. CD makes up to around 70% of all cases with endogenous Cushing’s syndrome but with an overall incidence of 1–2 per million per year and a prevalence of around 30 patients per million per year it is still a rare disease [2]. To this day, transsphenoidal removal of the tumor is still the therapy of choice, almost 80 years after Harvey Cushing used this procedure for the first time in the context of corticotroph pituitary adenomas [3]. Surgical resection results in remission in 65–90% of patients [4,5] but in case of persistent or recurrent disease therapeutic options are limited. One reason is that most of the drug treatments are aimed at the symptoms and not at curing the disease [6,7], and another one is that pathogenetics of corticotroph adenomas have remained obscure for a long time. Mainly due to material scarcity, only very small studies addressed gene mutations in CD, usually in the context of the main known endocrine familial genetic disorders. The conclusion was that CD arising in the context of, among others, McCune–Albright syndrome, multiple endocrine neoplasia or Carney complex account only for a negligible fraction of all CD [8,9,10,11]. This situation changed in the last few years, as access to reliable and relatively affordable high throughput sequencing lead to a multitude of disease specific genetic analyses for most of the known tumor entities. The first studies concentrated on the most widespread types of tumors and lead to the identification of so called “driver” mutations in these tumors (i.e., recurrent mutations associated with one or more types of tumors) [12]. The most comprehensive studies were performed in the context of big genetic consortia like The Cancer Genome Atlas (TCGA) that performed genetic analyses in over 30 types of cancer since its foundation in 2005 [13]. More importantly even, combining these types of genetic analyses with analyses of patient response to treatment lead to identification of somatic mutations that associate with therapy resistance like in the case of KRAS [14] and thus opened the way to new targeted therapies that are tailored to the genetic footprint of individual tumors. Even in the case of rarer diseases like CD, the combined effort of several reference centers for patient care and genetic analysis lead to important progress being made in the last few years. The breakthrough came in 2015 when we showed for the first time-using next generation exome sequencing-recurrent somatic mutations in a hotspot region of the Ubiquitin Specific Peptidase 8 (USP8) gene in CD [15]. Other studies using exome sequencing [16,17,18] or targeted sequencing [19,20,21,22,23] confirmed the fact that USP8 hotspot mutations are responsible for around half of the CD tumors and identified further recurrent mutations. We here performed a meta-analysis of all original works that are currently evaluable, aiming not only to assess the preponderance of all possible somatic driver mutations in CD but also to analyze the possible impact of these new mutations on tumorigenesis and therapeutic options in this rare disease.

## 2. Results and Discussions

### 2.1. Somatic Mutations in Deubiquitinases: The Most Frequent Recurrent Mutations in Cushing’s Disease

**Figure 1:**
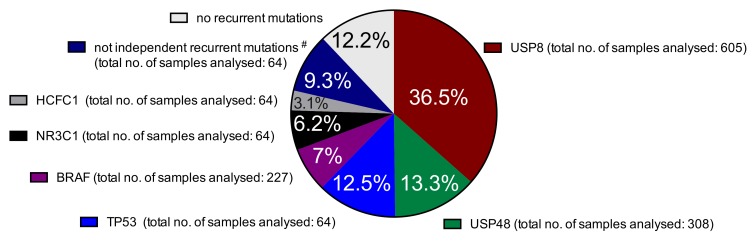
Incidence of all published recurrent somatic mutations associated with Cushing’s disease.

**Figure 2:**
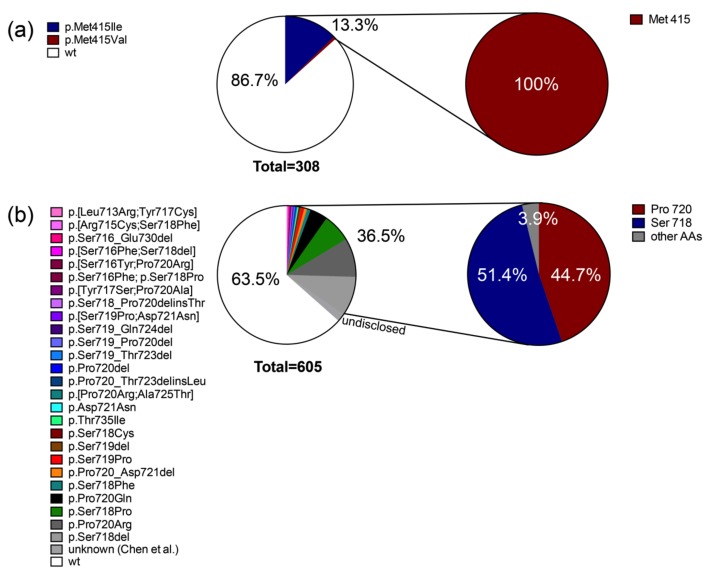
Summary and distribution of published USP48 (**a**) and USP8 (**b**) mutations in Cushing’s disease. On the left side pie-chart representations of percentages of all ACTH-producing pituitary adenomas carrying either wild-type (white) or different mutations (colored) found using both next-generation and Sanger sequencing and associated with CD published to date. On the right side pie-chart representations of the incidence of individual amino-acids affected by mutations reported in the same publications.

The 9 publications we analyzed are reporting together 605 individual CD cases, which is a remarkable number considering the rarity of this disease. Strikingly, from these samples, 221 (36.5%) of the CD tumors carried a somatic mutation in the USP8 gene (Figure 1). Together with the somatic mutations affecting the identical hotspot amino acid (p.Met415) in the USP48 gene in 13.3% (41/308) of the samples, the mutations in the two deubiquitinases account for about 50% of all known recurrent mutations in CD. Whereas the number of different mutations in USP48 is still limited (Figure 2a), for USP8 many variants have been described.

Interestingly, while the four mutations identified in the first two studies account for over 80% of the total number of mutations (p.Ser718del (*n* = 56), p.Pro720Arg (*n* = 55), p.Ser718Pro (*n* = 40), and p.Pro720Gln (*n* = 25), in the meantime more than 20 other different variants have been described with a prevalence of no more than 5 cases (Figure 2b). Of note, only two individual pathogenic variants (p.Asp721Asn and p.Thr735Ile; described for one case each) were completely outside the 14-3-3 binding motif (AAs 715-720). Their functional impact needs further investigation, as Reincke et al. have shown for the double mutation p.[Leu713Arg;Tyr717Cys] that only the mutation inside the 14-3-3 binding site (p.Tyr717Cys) has an impact on USP8 function but not the one outside the site (p.Leu713Arg) [15]. 

Several mutations affect complex sequences of more than one amino acid. Looking at the affected amino acids individually, the Ser718 (the phosphorylable serine in the 14-3-3 binding site) and Pro720 are affected in the majority of samples (together they are affected in 96.1% of all the samples carrying USP8 mutations) (Figure 2b). All the other amino acids put together are affecting only 11.7% (26/221) of samples. This apparent discrepancy between the prevalence is due to the fact that most of the other affected amino acids are part of complex mutations including always either Ser718 or Pro720, except for some of the Ser719 mutations and the two individual point mutations outside the 14-3-3 binding motif mentioned before (Asp721 and Thr735). Interestingly, Ser719, despite being nearer the phosphorylable Ser718 than Pro720, is affected in only 5.9% (13) of samples but as in more than 50% of those cases it occurs in complex mutations affecting more than one amino acid, only individual mutations in Ser219 affect barely 2.8% (6) of all samples (Figure 2b). This is however no surprise as it has been shown that both the phosphorylable serine (in the case of USP8, Ser718), and the proline in the position +2 (in the case of USP8, Pro720) are key to ensure 14-3-3 binding [24,25]. Their replacement with either a non-phosphorylable amino acid or with an amino acid other than proline, respectively, or their complete deletion leads to the inability of 14-3-3 proteins to bind USP8 and this in turn leads to increased deubiquitinating activity as also shown in humans [15]. We also showed that mutations in the Ser718 but also in Pro720 have the strongest effects on 14-3-3 binding and deubiquitinating power from all the mutations analyzed [15]. This does not mean that mutations in the other amino acids are irrelevant for the pathogenesis but probably they have more attenuated effect. The USP8 somatic hotspot mutations are very specific for ACTH secreting adenomas as the 11 non-functional ACTHomas carried the wild type USP8 only, as did the 121 GH producing adenomas (including 67 GHoma cases published separately by Ronchi et al. [26]), 60 prolactinomas and 60 inactive pituitary tumors that have been analyzed over time [15,16]. 

### 2.2. Prevalence of Different Other Recurrent Mutations in Cushing’s Disease

**Figure 3:**
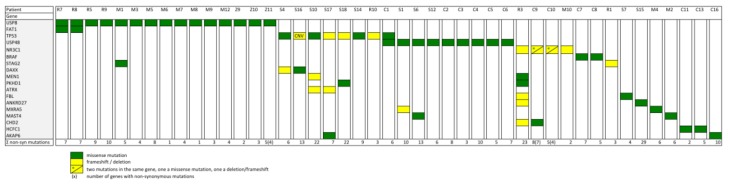
Schematic representation of all recurrent mutations, both independent and dependent, identified by next generation sequencing until now in Cushing’s disease. In the left column is the list of genes and in the upper line are listed the different samples in which the mutations have been identified. The letters are coding for the different studies R = Reincke et al. [15], M = Ma et al. [16], Z = Song et al. [27], S = Sbiera et al. [18], and C = Chen et al. [17], while the numbers indicate the individual samples as listed in the original paper.

In addition to the hotspot USP8 and USP48 mutations, our meta-analysis identified multiple other recurrent mutations. We have found different inactivating somatic mutations in TP53 in 12.5% (8/64), the same hotspot somatic mutation p.Val600Gln in BRAF in 7% (16/227), 6 different somatic mutations in the glucocorticoid receptor (GR/NR3C1) in 6.2% (4/64) and mutations in HCFC1 in 3.1% (2/64) of the CD adenoma samples analyzed (Figure 1 and Figure 3). 

There were also other genes with somatic mutations in more than one sample. However, most of these mutations occurred at least in one of the samples concomitantly with one of the more prevalent driver mutations. For example, both recurrent FAT1 mutations occurred in cases carrying already USP8 mutations while the recurrent DAXX and ATRX mutations in samples with TP53 pathogenic variants. Other genes carried somatic mutations in one sample concomitantly and in one sample lacking one of the more widely mutated genes as in the case of FBL, ANKRD27 etc. (Figure 3). Considering also these latter samples, the percentage of samples without any recurrent mutation is only 12.2% (Figure 1), a dramatically low percentage taking into consideration that 5 years ago almost no driver mutations for CD were known. Of course, this percentage is probably not reflecting the reality, as the cohort size for the various analyses differed significantly. This is due to the fact that not all the samples have been analyzed throughout by exome sequencing. Actually, most of the samples have been only sequenced for certain genes or even only for certain regions of a gene after an incipient discovery of possible candidates by exome sequencing. Examples for this bias are TP53 or BRAF mutations that have been described almost exclusively in one study but not in the others [17,18]. The best solution to overcome this bias is to further increase the number of samples studied by next generation sequencing through large scale international collaborations. An alternative is to count only samples analyzed by exome sequencing. When we applied this strategy, the frequency of the different mutations is slightly different (see Supplementary Figure S1). However, this evaluation is also biased against USP8 as except the first two studies [15,16], the subsequent studies have chosen USP8 wt samples only for their next generation sequencing.

### 2.3. Pathogenetic Relevance of the Most Frequent Mutations in Cushing’s Disease

**Figure 4:**
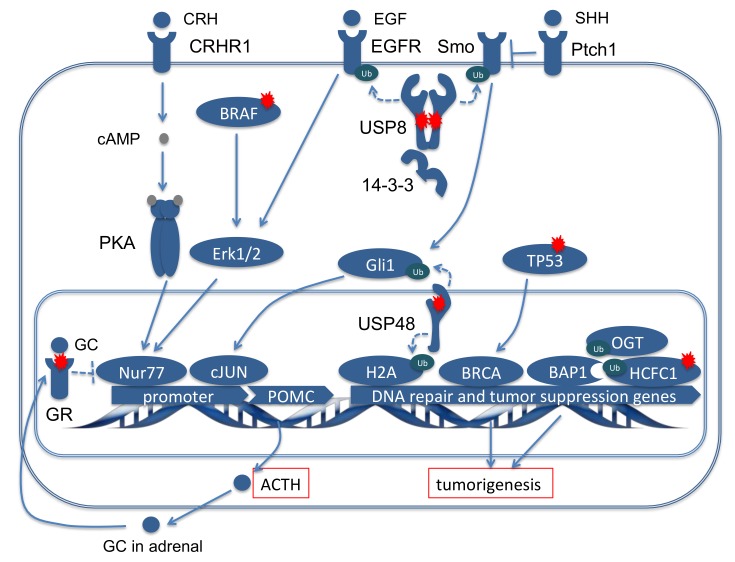
Graphic representation of all independent recurrent mutations in the context of a corticotroph pituitary cell in Cushing’s disease. In blue are represented the different proteins and factors that are involved in different processes in a cell. The independent recurrently mutated genes are marked with red sparks. The interactions are based on literature and should visualize direct and indirect involvement of the mutations in the cellular processes in a Cushing’s pituitary cell. Doted arrows indicate hypothesized mode of action of these mutations.

The high frequency of mutations in deubiquitinase genes already indicates that classical oncogenes or tumor suppressor genes might be less important than for other tumors, whereas ubiquitination and deubiquitination seems to be quite relevant. However, we recently identified somatic TP53 mutations [18], and another group described mutations in BRAF as potential driver mutation [17]. In the following sections, we will discuss the role of the different mutation in the development of CD (Figure 4).

#### 2.3.1. Deubiquitinases, the Key Driver Genes in CD

When the results of the next generation sequencing results of CD tumors were first published, it came with surprise that a gene that was never associated with pituitary tumors was the most often mutated gene in CD, namely USP8 [15]. Since that time ubiquitination and dubiquitination of protein disposal/recycling are in a new spotlight of endocrinologists/oncologists. Five years and several studies later, we and others have shown that USP8 is not the only deubiquitinase mutated in CD, but has been joined by USP48. Recurrent mutations in these two genes are responsible for half of all the corticotroph tumors. Of note, no single sample carried both USP8 and USP48 mutations suggesting that they are mutually exclusive. Also, the tumors with USP8 and USP48 mutations were significantly smaller, while the carriers were also generally younger, suggesting that these mutations might lead to less aggressive tumors, but probably with a more efficient ACTH secretion. The ultimate evidence that USP8 plays an important physiological role in the corticotroph pituitary cells and that its mutations are involved specifically in the pathogenesis of CD is the discovery of a de-novo germline mutation in the hotspot region of this gene (p.Ser719Pro) in a young female patient suffering from CD [28]. The germline mutation USP8 mutation was not lethal despite pregnancy complications, however, this mutation led to development of a corticotroph tumor at puberty that then induced the typical biochemical and morphological manifestations of Cushing’s syndrome. 

How do mutations in USP8 and USP48 act in CD? Ubiquitination is the covalent attachment of a small protein modifier ubiquitin to a substrate protein and is ubiquitous in virtually all cellular processes hence the name [29]. Aside from proteasomal degradation, ubiquitination plays important roles in transcriptional regulation, protein trafficking, including endocytosis and lysosomal targeting, and activation of kinases involved in signaling processes [30]. It is counteracted by the reversed process called deubiquitination, which leads to protein recycling or to protein trafficking in the reverse direction [31]. So what is the role of this process in CD and what do the mutations in USP8 and USP48 do? As mentioned previously, the USP8 is affected by several different mutations in the same hotspot in the 14-3-3 binding motif while USP48 has only one amino acid affected in the catalytic domain of the protein. Why this difference in localization? Structural studies [32] have shown that the catalytic domain of USP8 is normally in a closed conformation and cannot excise ubiquitin molecules except after a conformational change induced by controlling molecules so a mutation in the catalytic subunit directly would not affect gravely the function of the protein. USP48 however has a more open catalytic subunit therefore a mutation in this region would affect permanently the activity of this enzyme. Wherever the mutation locus, the mutations in both enzymes lead to increased deubiquitination activity of these proteins [15,17,18]. This activity is directed most likely against specific substrates. For USP8, the best-studied substrate is EGFR, which is then recycled and re-pushed in the system. Increased EGFR activity results in ERK1/2 activation [33] which, in a parallel manner to the physiological pathway involving the CRHR stimulation by CRH followed by cAMP dependent PKA activation-lead to activation and binding of the nuclear receptor Nur77 to the Nur responsive element in the promoter of POMC leading to an increased ACTH secretion [15,34]. However, EGFR is not the only substrate of USP8 in CD, recent studies showed several proteins deregulated in the context of USP8 mutations making them putative substrates of USP8 in CD and all with a possible role in either ACTH production or cell cycle [34,35]. Most importantly, it has also been shown in other diseases that USP8 deubiquitinates SMO in the SHH signaling pathway and this mechanism might be relevant in CD as well [36]. USP48 on the other hand has been shown to deubiquitinate Gli1 and histone H2A in CD. SHH pathway has long been shown to regulate CRH signal transduction in the pituitary cells [37]. The subsequent recycling of Gli1 induced by mutated USP48 hyperactivation leads then to both ACTH secretion and H2A deubiquitination to promote tumor growth [18,38] (Figure 4).

Can we target USP8 and USP48 therapeutically? Since half of all CD tumors are affected by mutations in these genes, deubiquitinases might be indeed an attractive target for therapies. In the case of USP8 there are some inhibitors that have been already tested in vitro in other cancers that were resistant to EGFR inhibitors [39]. As EGFR is the substrate most often reported for USP8 and preliminary reports show EGFR overexpression and sensitivity to EGFR inhibitors like gefitinib in primary CD tumor cells [16], these inhibitors might also be an option for the treatment of USP8 mutated CD tumors [40]. For USP48 unfortunately there are no inhibitors developed yet. Addressing molecules with ubiquitous roles like the histone H2A is also not a viable option, however, targeting the sonic hedgehog pathway for anti-cancer treatment is an emerging concept [41]. 

#### 2.3.2. Classical Oncogenes and Tumor Suppressor Genes

As mentioned above, until recently, mutations in oncogenes or tumor suppressor genes were unknown in CD. However, our meta-analysis suggests that TP53 mutations occur in about 12% of ACTH-producing pituitary tumors. The tumor suppressor gene TP53 has been studied for a long time in many cancers and germline mutations in TP53 causing oncogenic syndromes like the Li-Fraumeni syndrome [42,43]. At somatic level TP53 is the most frequently mutated gene with more than 50 percent of human tumors containing a somatic pathogenic variant or deletion of the *TP53* gene [44]. Loss of p53 creates genomic instability that most often results in further mutations and increased tumorigenesis [45]. The majority of the TP53 mutations in the CD were described in our recent exome analysis [18]. All of them were inactivating mutations. Most were frameshift mutations that introduced stop codons while one was a deletion of a big fragment of the chr17 at the location of the TP53 gene consistent with a probable copy number loss (chr17:g.7576876-7577303del). In the remaining CD, two of the point mutations were already described as pathogenic mutations in a germline setting (rs28934575 and rs28934576) and the third affected Asp208, which (as part of the S6-S7 loop of p53 DBD) is modulated by the interaction with DNA and is also a regulatory site for p53 transcription-independent functions [46]. However, to promote tumorigenesis both alleles of TP53 have to be altered. Alternatively, a second hit might involve other genes in the TP53 pathway. We hypothesize that TP53 mutations in CD lead to interference in DNA repair mechanism through BRCA1 [18] (Figure 4) or regulation of apoptosis. The latter is also suggested by the presence of concomitant recurrent mutations in ATRX (a gene silencing protein) and DAXX (Death Domain Associated Protein) [47,48] (Figure 4). Interestingly, the TP53 mutated CD tumors were larger in size than the average and were also generally more aggressive. Preclinical and first clinical studies are testing TP53 as therapeutic target in other cancers. [49,50]. While awaiting the preliminary results of these studies the pituitary community will have to ascertain the full incidence rate of TP53 somatic mutations in CD tumors. Until now, these mutations have been described only after next generation sequencing which is still a quite expensive technology when widely applied. A systematic analysis of TP53 status in a high number of samples has been impeded by the fact that the mutations are distributed over the whole gene. Thus, Sanger sequencing of the complete reading frame would require an appropriate amount of DNA material that is often not available.

The other recurrently mutated gene in CD that one immediately associates with tumorigenesis is, as mentioned before, BRAF. Surprisingly enough, the same mutation in BRAF (p.Val600Gln) was identified in all the reported samples; this mutation is mostly associated with very aggressive forms of cancer [51,52] and not, like in the case of CD, with benign tumors. This is particularly true as the BRAF mutated CD tumors do not appear to be bigger or associated with more malignant clinical features as the wild-type tumors [17]. What has been shown was an elevated kinase activity of BRAF V600E compared to wild-type BRAF in corticotroph adenomas leading to activation of MAPK pathway involving the ERK1/1 and transactivation of POMC, leading to increased ACTH production [17] (Figure 4). Interestingly, the same study has also addressed the therapeutic potential of BRAF inhibitors in treating CD by showing that treatment with vemurafenib of primary cultured human corticotroph adenomas lead to a concentration dependent decrease of ACTH production in both BRAF wild-type and mutated tumors but with a more dramatic decrease in the mutated ones. There was unfortunately no data on the effect of vemurafenib on cell viability or proliferation in the same cells.

#### 2.3.3. Alterations in the Glucocorticoid Pathway

In the case of the GR mutations the connection with CD is quite obvious, as the GR is involved in the regulation of ACTH production in the corticotroph cells. Under physiological conditions, CRH secreted by the hypothalamus binds its receptor, a G-coupled protein receptor, on the corticotroph cells, which then induces cAMP production and activation of protein kinase A that afterwards activates Nur77, that in turn binds the Nur responsive element in the promoter and induces transcription of ACTH precursor POMC leading to ACTH secretion [53]. ACTH circulates to the adrenal glands, where it induces glucocorticoid secretion (GC). The ACTH production is not only controlled by the CRH production, which usually follows a circadian rhythm, but there is also a feedback mechanism in place, in which the adrenal GCs bind the GR in corticotroph cells that than block the POMC promoter through a trans-repression of Nur77 (Figure 4). Of course if the GR in these cells is defective or truncated (as it seems to be the case in subjects with CD, in whom GR alterations are frameshift mutations, deletions, or even double mutations including a missense mutation in the ligand binding domain), the feed-back mechanism is disrupted and the cells become glucocorticoid resistant [54,55]. The latter certainly results in an increased production of ACTH but theoretically an increasing number of corticotroph cells may also be induced (either through an aggravated differentiation of lineage precursor cells or through decreased apoptosis, as the organism tries to cope with inadequate levels of ACTH). This effect may be in a way similar to the occurrence of pituitary hyperplasias [56,57]. This is not a proven theory yet but it might be an interesting direction of research especially now as mutations of the GR are associated with CD. Experiments in rats have shown that the full function of a defective GR could be restored by expression of cloned receptor cDNA [58] and there are some promising experiments involving selective glucocorticoid receptor modulators (SEGRAs) [59], however none of them have been tested in the pituitary cells. Furthermore, the number of CD tumors affected by somatic GR defects is quite low, at least for the moment, not justifying an increased effort in addressing this therapeutic avenue for the moment.

### 2.4. Functional Network Analysis of the Genes Mutated in ACTH Producing Pituitary Adenomas

Looking at the pathways involving the recurrently mutated genes (Figure 4), it appears that they are partially interconnected. The recurrent mutations are however not the only mutations affecting CD. There are 385 more genes mutated in only one of the samples analyzed (Supplementary Figure S2). 

**Figure 5:**
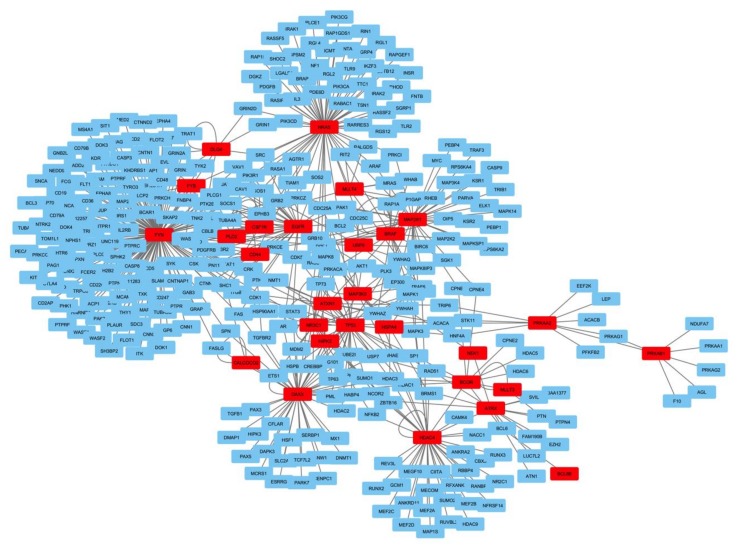
Bioinformatically engineered network representation of densely connected cluster nodes in CD having the highest impact on tumorigenesis. In red are represented mutated genes found in CD and in blue the main interaction partners in the cluster network (365 nodes, 576 edges) are given.

A network analysis of all the genes with reported mutations in the ACTH producing pituitary adenomas, both recurrent and unique, revealed that with a few exceptions the mutations affect proteins that are one way or the other interconnected (Supplementary Figure S3). Looking at the clusters that have the strongest impact on the whole network topology we found four densely connected cluster regions: cluster 1 involved MAP2K1 (mutated), BRAF (mutated), and RAF1; cluster 2 HRAS (mutated), FYN (mutated), and VAV1; cluster 3 PRKAA2 (mutated), PRKAB1 (mutated), and PRKAG1; cluster 4) HDAC4 (mutated), DAXX (mutated), BCOR (mutated), ATRX (mutated), USP7, and BCL6 (Figure 5). 

Central neighbors to these four cluster nodes were USP8 with 14-3-3 proteins like YWHAQ (connected also to BRAF), YWHAG (connected also to BRAF/HDAC4/TP53) and TP53. USP48 does not appear in this network, however this is due to the relatively limited amount of studies addressing this protein in humans, and we therefore did not find experimentally validated interactions.

**Figure 6:**
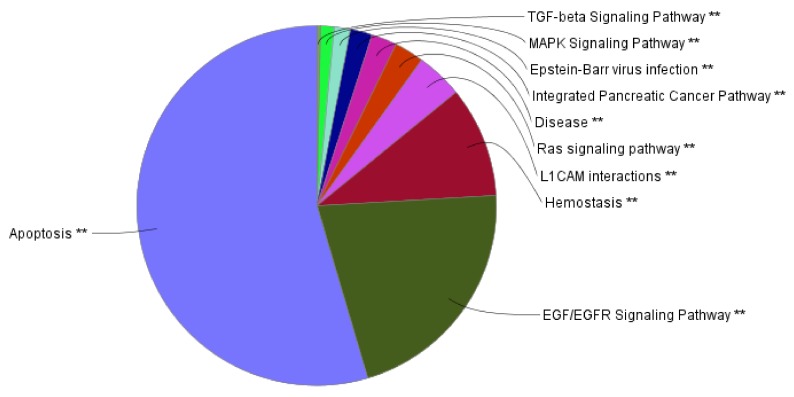
Pie chart representation of the preponderance of highly enriched processes and pathways in the CD based on an analysis of 365 densely connected cluster genes found in CD. For the complete list of all the enriched pathways of the connected cluster genes in CD please see Supplementary Table S1. ** indicates significant GO terms and pathways with p-value <0.05.

Looking at the pathways in which the 365 densely connected cluster genes are involved, the lions’ share are pathways involved in apoptosis as opposed to cell cycle disturbances as one would see in more malignant cancer, the EGF/EGFR signaling involving not only USP8 but also several of the uniquely mutated genes (Figure 6, Supplementary Table S1) suggesting that not only the recurrently mutated genes are important for the tumorigenesis in CD but that through converging and interconnected pathway all the mutations affecting CD play a role. This functional pathway analysis is highly significant with *p* < 5 × 10^−16^. 

## 3. Materials and Methods

### 3.1. Patient Cohorts

All published data clearly suggest that germline mutations causing CD are extremely rare and account only for a negligible fraction of all cases with CD [8,9,10,11]. Accordingly, in this analysis we did not address this topic as it was already analyzed in more detail elsewhere [56].

For this meta-analysis we screened in Pubmed for all publications reporting somatic mutations associated with pituitary Cushing’s disease. Following the first description of recurrent USP8 mutations in 2015 by our group [15] there have been several publications over the years, analyzing the presence of somatic driver mutations not only in adult and pediatric CD but also in tumors associated with Nelson’s syndrome by the use of both next generation exome and Sanger hotspot sequencing [16,17,18,19,20,21,22,23,27,57]. In this meta-analysis, we analyzed all individual samples contained in these 10 publications. We did not differentiate between tumors with and without Nelson’s syndrome as the study of Perez-Rivas et al. did not demonstrate a relevant difference in the frequency of driver somatic mutations in these two types of tumors [23]. For our analysis we used data resulting from exome sequencing reported by Reincke et al. [15], Ma et al. [16], Song et al. [27], Chen et al. [17], and Sbiera et al. [18]. Exome sequencing data were further filtered to exclude intronic and synonymous variants as these variants would not influence protein function. From the study by Song et al. we used only the data from three of the 20 patients with available exome results, because 17 have been included in two of the other analyzed studies [16,17]. The reason we selected the data of the study by Chen was that a detailed analysis suggested to us that this study was slightly more comprehensive, including more non-synonymous mutations per sample as in the other two studies reporting the same results.

To have a better idea about the frequency of the pathogenic variants we have also integrated the targeted sequencing data from the five studies mentioned above as well as from the studies that have analyzed specific frequent somatic driver mutations in CD using Sanger sequencing [20,22,23,57]. Here we were confronted with another problem as the cohort analyzed by Sanger sequencing reported by Ma et al. was completely included in the bigger cohort analyzed by Chen et al. However, the later study did not report the frequency of the individual mutations in the USP8 gene. Therefore, we used the numbers from Chen et al. for our analysis of the frequency of all mutations in a certain gene but we used the study by Ma et al. for reporting the frequency of individual mutations in USP8. We also excluded the publication by Faucz et al. [19] from any of our analyses as the number of cases carrying the specific mutations was not revealed and 24 of the 42 cases have been included in another study without us having the possibility to separate new from already reported samples [22].

### 3.2. Bioinformatics Analyses

Bioinformatics analysis combines network reconstruction and functional analysis (method see https://www.ncbi.nlm.nih.gov/pubmed/29797762). We reconstructed a network based on all mutations identified in CD by exome sequencing in all the publications analyzed (607 Genes), and their direct interaction partners (neighbors) from the Human Protein Reference Database (HPRD; binary protein–protein interactions, 9620 proteins (nodes) and 39,185 protein–protein interactions (edges), release 9 from 13 April 2010). The network contains 2100 nodes and 2802 interactions. The network was visualized using the Cytoscape software version 3.4.0 [60]. Next, we analyzed the reconstructed network for functional clusters (modules) using the Cytoscape plugin MCODE ([61]; standard parameter). We further performed a functional pathway enrichment analysis using the Cytoscape plugin ClueGO [62]; parameters: GO term grouping, *p*-value < 5 × 10^−16^). 

## 4. Conclusions

Our meta-analysis and recently performed experiments clearly show that CD tumors harbor somatic driver mutations in several genes, from which USP8 is the most prevalent. Together with recurring somatic mutations in USP48, TP53, BRAF, and NR3C1 these mutations account for development of CD tumors in over 70% of the patients. Furthermore, an integrated network analysis of all mutations described for CD tumors revealed that despite the mutational variability there might be a mechanistical convergence through common oncogenic pathways.

## Supplementary Materials

The following are available online at https://www.mdpi.com/2072-6694/11/11/1761/s1, Figure S1: Alternative incidence of published recurrent somatic mutations associated with CD when considering only the results from next generation sequencing, Figure S2: Schematic representation of all non-synonymous mutations identified by next generation sequencing until now in CD, Figure S3: Reconstructed whole network topology of the 607 CD genes and their direct interaction partners (2100 nodes and 2802 edges; mutations in red), Table S1: List of all the most significantly enriched processes and pathways due to 365 densely connected cluster genes in CD.
