# Supplementary material for: The New Genetic Landscape of Cushing’s Disease: Deubiquitinases in the Spotlight
Source: Cancers (Basel). 2019 Nov 8;11(11):1761. doi: 10.3390/cancers11111761 (PMC6895825; doi:10.3390/cancers11111761)
Supplement: Supplementary file 1 [file cancers-11-01761-s001.zip › cancers-621823 - supplementry - final/cancers-621823 -supplementary - final.pdf]

Supplementary Data: The New Genetic Landscape of Cushing's Disease: Deubiquitinases in the Spotlight

Silviu Sbiera, Meik Kunz, Isabel Weigand, Timo Deutschbein, Thomas Dandekar and Martin Fassnacht

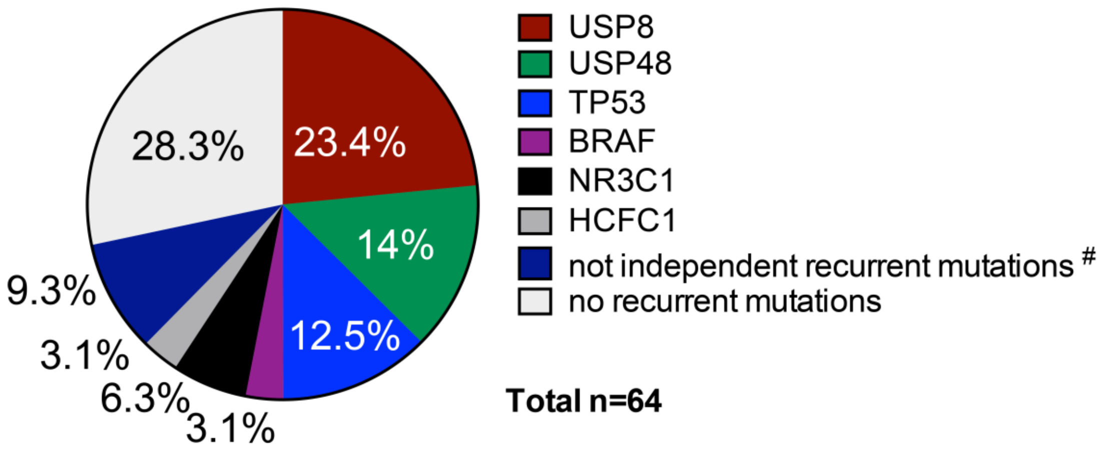

Figure S1. Alternative incidence of published recurrent somatic mutations associated with Cushing's disease when considering only the results from next generation sequencing.

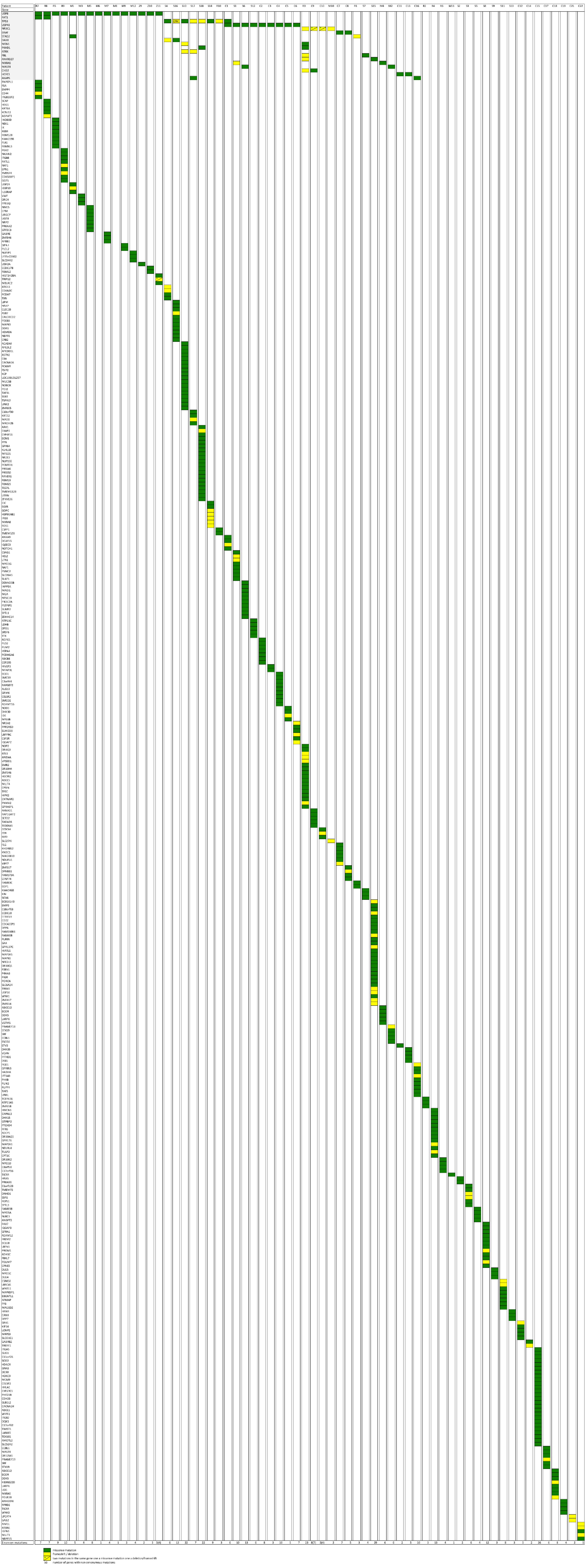

Figure S2. Schematic representation of all non-synonymous mutations identified by next generation sequencing until now in Cushing's disease.

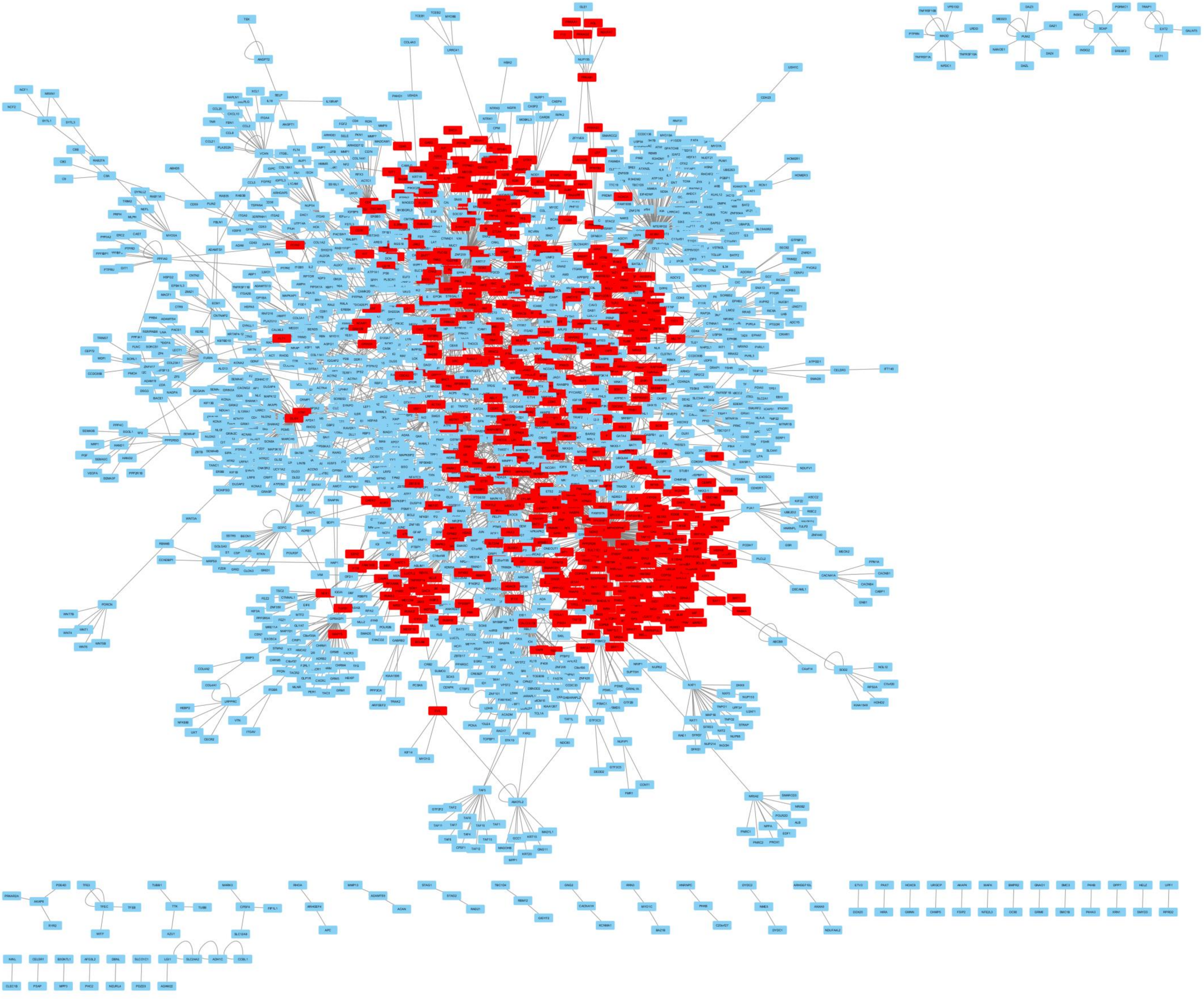

Figure S3. Reconstructed whole network topology of the 607 CD genes and their direct interaction partners (2100 nodes and 2802 edges; mutations in red).

Supplementary Table S1. See separate file.

© 2019 by the authors. Licensee MDPI, Basel, Switzerland. This article is an open access article distributed under the terms and conditions of the Creative Commons Attribution (CC BY) license (http://creativecommons.org/licenses/by/4.0/).
